# Longitudinal changes in macular curvature and axial elongation in school children

**DOI:** 10.1186/s40942-025-00752-8

**Published:** 2025-11-25

**Authors:** Mikiko Honbou, Takehiro Yamashita, Hiroto Terasaki, Ryo Asaoka, Naoya Yoshihara, Naoko Kakiuchi, Taiji Sakamoto

**Affiliations:** 1 Department of Ophthalmology, Kagoshima University Graduate School of Medical and Dental Sciences, Kagoshima, Japan; 2 Department of Ophthalmology, Seirei Hamamatsu General Hospital, Shizuoka, Japan; 3 Seirei Christopher University, Shizuoka, Japan; 4 Nanovision Research Division, Research Institute of Electronics, Shizuoka University, Shizuoka, Japan; 5 The Graduate School for the Creation of New Photonics Industries, Shizuoka, Japan

**Keywords:** Macular curvature, Axial elongation, School myopia

## Abstract

**Background:**

To investigate the relationship between changes in macular curvature and axial elongation in school children.

**Methods:**

This prospective cohort study involved 75 right eyes of elementary school students for six years (from 8 to 9 to 14–15 years). In the first and last year, all participants underwent optical axial length measurement and color fundus photographs. Optical coherence tomographic vertical cross-sectional images of the macula were taken and used for the analysis. The macular curvature was plotted as the retinal pigment epithelium and fitted to a second-degree polynomial equation using ImageJ software to calculate the macular curvature. Wilcoxon’s signed rank test was used to compare macular curvature and axial length. The association between axial elongation and macular curvature was investigated using Spearman’s correlation.

**Results:**

The mean axial length in the last year (24.82 mm) was significantly greater than that in the first year (23.34 mm). Likewise, the mean macular curvature was significantly greater in the last year (0.041) than in the first year (0.014). Macular curvature in the first year was significantly negatively correlated with axial elongation over 6 years (*r*=-0.28, *p* = 0.014). The change in macular curvature was significantly positively correlated with axial elongation (*r* = 0.28, *p* = 0.017).

**Conclusions:**

During the period from 8 to 9 years to 14–15 years of age, the macular curvature increased, and eyes with smaller macular curvature at 8–9 years tended to have greater axial elongation. Macular curvature can be a predictor of axial elongation during this period.

## Background

**Fig. 1:**
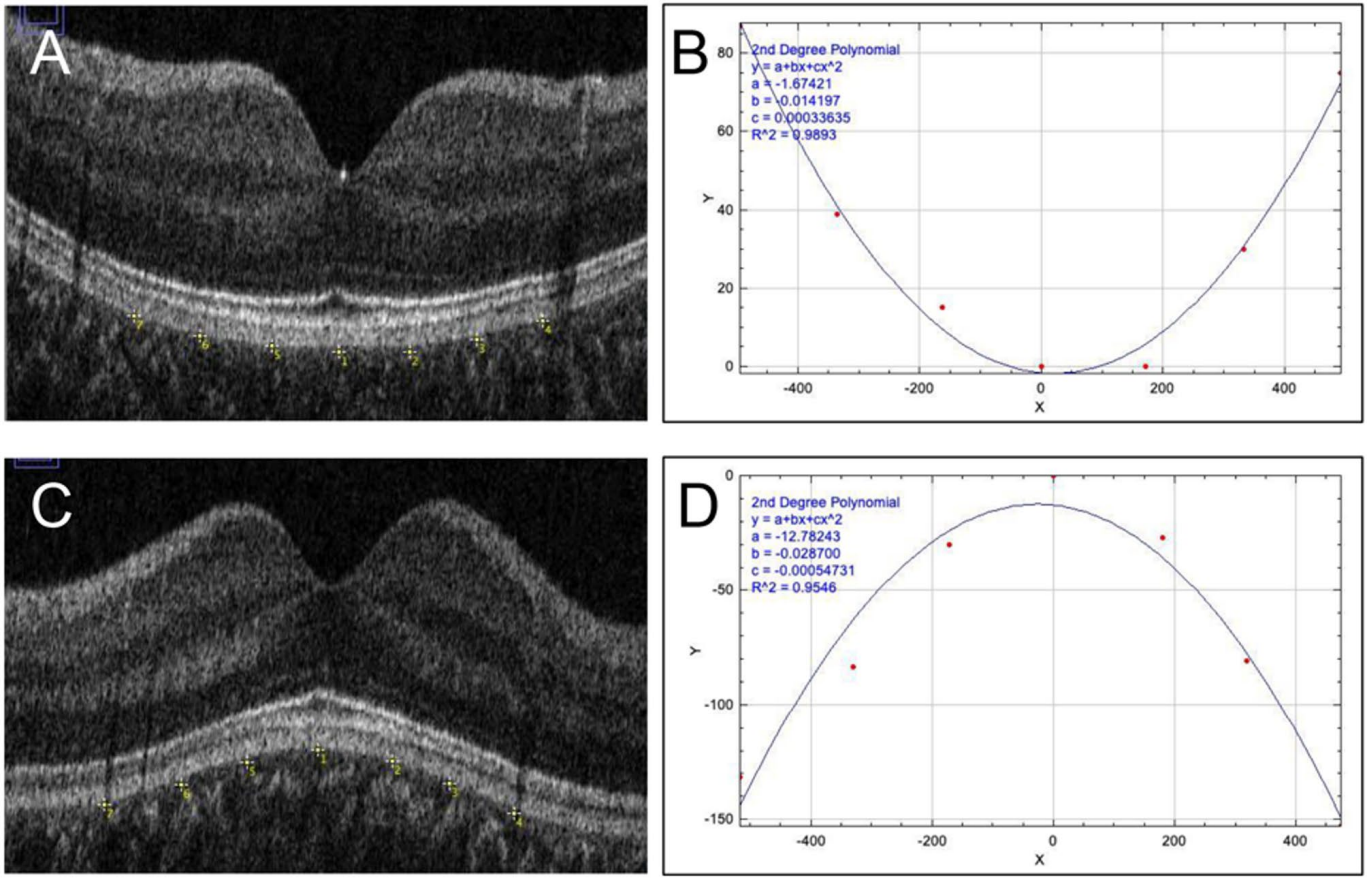
Quantification of Macular Curvature. To evaluate macular shape, the vertical OCT cross-sectional image was first magnified fourfold in the vertical axis and then rotated to achieve symmetry of the ellipsoid zone around the fovea. Subsequently, the retinal pigment epithelium (RPE) line was manually traced at seven distinct points located within ± 1500 µm from the foveal center (**A**, **C**). The original OCT image coordinates (‘x’, ‘y’) were then transformed into a new coordinate system, where the RPE at the foveal center was set as the origin. These converted points were fitted to a second-degree polynomial function, specifically: ax²/100 + bx + c, using the curve fitting tool in ImageJ. In this context, a positive value of ‘a’ indicates a convex macular profile (**B**), while a negative value corresponds to a dome-shaped configuration (**D**)

The retina generally curves slightly outward to conform to the roundness of the eyeball. However, divergent macular structures, such as staphyloma and dome-shaped macula, have been reported in adult eyes [1–7]. Macular curvature varies from person to person and is classified into convex, dome, and flat types on the vertical cross-sectional images of optical coherence tomography (OCT) [8]. The degree of macular curvature can be quantified by plotting the retinal pigment epithelial cell layer of the macular area, approximating it to a quadratic curve, and using the coefficient of x^2^ [9, 10]. Using this method, the larger the coefficient of x^2^, the steeper the curve, and the smaller the coefficient, the flatter the curve. Convex types have positive values, dome types have negative values, and flat types will have values ​​close to zero (Fig. 1). A study of adult macular curvature at the UK Biobank reported that greater degrees of myopia are associated with steeper macular curvature, and dome types are more common in hyperopic eyes [9].

Considering that there are no long-term follow-up studies of macular curvature, changes in macular curvature during growth remain unknown. A cross-sectional study reported steeper macular curvature in children aged 12–13 years than in those aged 8–9 years. Further, at 8–9 years of age, no meaningful correlation was found between axial length and macular shape, but a significant positive correlation was observed between the two at 14–15 years of age [10]. This suggests that macular curvature changes during school age are related to axial elongation, but it remains merely speculative, as this was not a long-term observational study. Additionally, we speculated that macular curvature might be a predictor of axial elongation.

Myopia mainly progresses due to axial elongation during school age [11, 12]. Several reports on the predictors of axial length in children exist. Birth weight and axial length have been reported as predictors of axial length at ages 3–6 [13, 14], while parental myopia and axial length–corneal radius ratio have been reported as predictors of axial length at ages 6–9 [15]. In a cohort study of school-age children, eyes with longer axial length at baseline had larger axial elongation [16, 17]. In infants, eyes with longer axial length at birth had smaller increases in axial length at 3 years [14]. The relationship between axial length at baseline and axial elongation is reversed between infants and school-age children. Furthermore, several studies have reported predictors for the initiation and advancement of myopia [18]. Several ocular biometric parameters assessed in the first year of life—including axial length, refraction, anterior chamber depth, lens and vitreous chamber thickness, and corneal curvature—have been reported as strong predictors of later refractive outcomes and axial length [16, 19]. In addition to these anatomical measures, lifestyle-related factors such as time spent on near work, frequency of outdoor activities, and level of education have also shown significant associations with refractive development [18, 20]. Furthermore, demographic and personal characteristics—including age, sex, ethnicity, stature, baseline visual acuity, and parental refractive status—have been recognized as contributing variables in the progression of myopia [21, 22]. Myopia progresses during school age as the eyeball expands to become oval-shaped anteroposteriorly, and the focus shifts in front of the retina [23, 24]. Oval eyeball expansion leads to steeper macular curvature (more convex) and is expected to affect the degree of axial elongation. Therefore, macular curvature may be a promising predictor of axial elongation. However, there is a lack of long-term studies investigating changes in macular curvature and axial length.

While cross-sectional studies include generational and individual differences in addition to age-related changes, long-term observational studies can directly capture true age-related changes. Therefore, this study sought to investigate the relationship between changes in macular curvature and axial length in school children through long-term observational follow-up.

## Methods

### Ethics statement

All study procedures were carried out in line with the ethical guidelines of the Declaration of Helsinki and were authorized by the IRB of Kagoshima University Hospital (Approval No. 170116(643)). Prior to participation, written informed consent was secured from all subjects and their respective guardians. This study has been officially registered with the University Hospital Medical Information Network Clinical Trials Registry (UMIN-CTR) under the registration number UMIN000015239.

### Participants

This longitudinal, prospective, observational study involved third-grade students aged 8–9 years at the time of the initial examination [16]. The participants were enrolled at the Elementary School affiliated with the Faculty of Education, Kagoshima University. Among the 144 third-grade students who were eligible to participate, 122 (87.4%) and their parents provided written informed assent and consent. The examinations were conducted between November 17 and December 18, 2014, in the first year (mean age: 8–9 years), and during the same period in 2020, in the final year (mean age: 14–15 years). To avoid artificially narrow confidence intervals and low P-values, only the right eyes were included in the analysis.

Exclusion criteria included pathological findings observed on fundus photography; systemic diseases affecting the eyes; a history of abnormal development; previous ocular trauma or surgery; poor cooperation from the child; absence from follow-up due to prolonged absenteeism or school transfer; unclear visualization of the peripheral fundus; and unmeasurable ocular or fundus parameters.

Fourteen eyes were excluded due to difficulties in measuring fundus or ocular parameters, and due to absenteeism, school transfer, or attending a different junior high school; 33 students were not included in the final analysis.

As a result, data from 75 students were included in the final analysis, covering their third-grade year in elementary school (age 8–9) and third-grade year in junior high school (age 14–15).

Color fundus photographs and vertical cross-sectional OCT images were obtained using a 3D OCT-1 Maestro device (Topcon, Tokyo, Japan). The images were acquired as a 3D 6 × 6-mm macular cube scan. Since the image size decreases with increasing axial length, a built-in magnification correction function in the OCT system was applied to adjust for this effect [25]. The repeatability and reproducibility measurements by the 3D OCT-1 Maestro macular cube scans were excellent [26]. Therefore, the OCT scan was performed in a single measurement. Additional scans were acquired by the operator if the scan quality was determined to be unacceptable. To minimize variability due to misplacement of the measurement location and/or segmentation error, an experienced researcher (T.Y.) checked the locations of the fovea in the center of the macular vertical cross-sectional scans. Axial length measurements were performed separately with an OA-2000 optical biometer (Tomey, Nagoya, Japan).

### Measurement of macular curvature

We quantified macular curvature by magnifying OCT images and fitting the resulting data to a second-degree polynomial equation, in accordance with earlier studies [10]. As an initial step, the vertical OCT cross-section was enlarged by a factor of four along the vertical axis to improve visualization of macular shape. The image was subsequently processed using ImageJ software (NIH, Bethesda, MD), a widely used tool for image analysis in biomedical research, and adjusted to achieve symmetrical alignment of the perifoveal ellipsoid zone.

Next, the retinal pigment epithelium (RPE) line was manually marked at seven locations within ± 1500 μm from the foveal center. The original x and y coordinates from the OCT image were converted into a new coordinate system, with the foveal RPE position set as the origin.

The converted coordinates were fit to the following quadratic equation using the curve fitting function in ImageJ.:

$$\:y=\frac{a{x}^{2}}{100}+bx+c$$

Here, *a*, *b*, and *c* represent coefficients determined by the least squares fitting method in ImageJ. Under these conditions, a positive **a** value indicates a convex macula (Figs. 1A, B), while a negative **a** value indicates a dome-shaped macula (Figs. 1C, D). The **a** coefficient was used as a quantitative index of macular curvature.

### Statistical analyses

Statistical analyses were conducted using IBM SPSS Statistics version 21.0 (Windows platform; Armonk, NY, USA). To evaluate the inter-rater consistency of optic disc measurements, intraclass correlation coefficients (ICCs) were calculated based on a two-way mixed-effects model assuming absolute agreement. Alterations in axial length and macular curvature from ages 8–9 to 14–15 were statistically assessed using the Wilcoxon signed-rank test, a nonparametric method for paired samples. The correlations between axial length and macular curvature at 8–9 and 14–15 years or their changes were analyzed using Spearman’s correlation. Partial correlation analysis between axial elongation and macular curvature in the first year was used to adjust the effect of the first-year axial length. *P* < 0.05 denoted statistical significance.

## Results

### Ocular parameters of participants

**Fig. 2:**
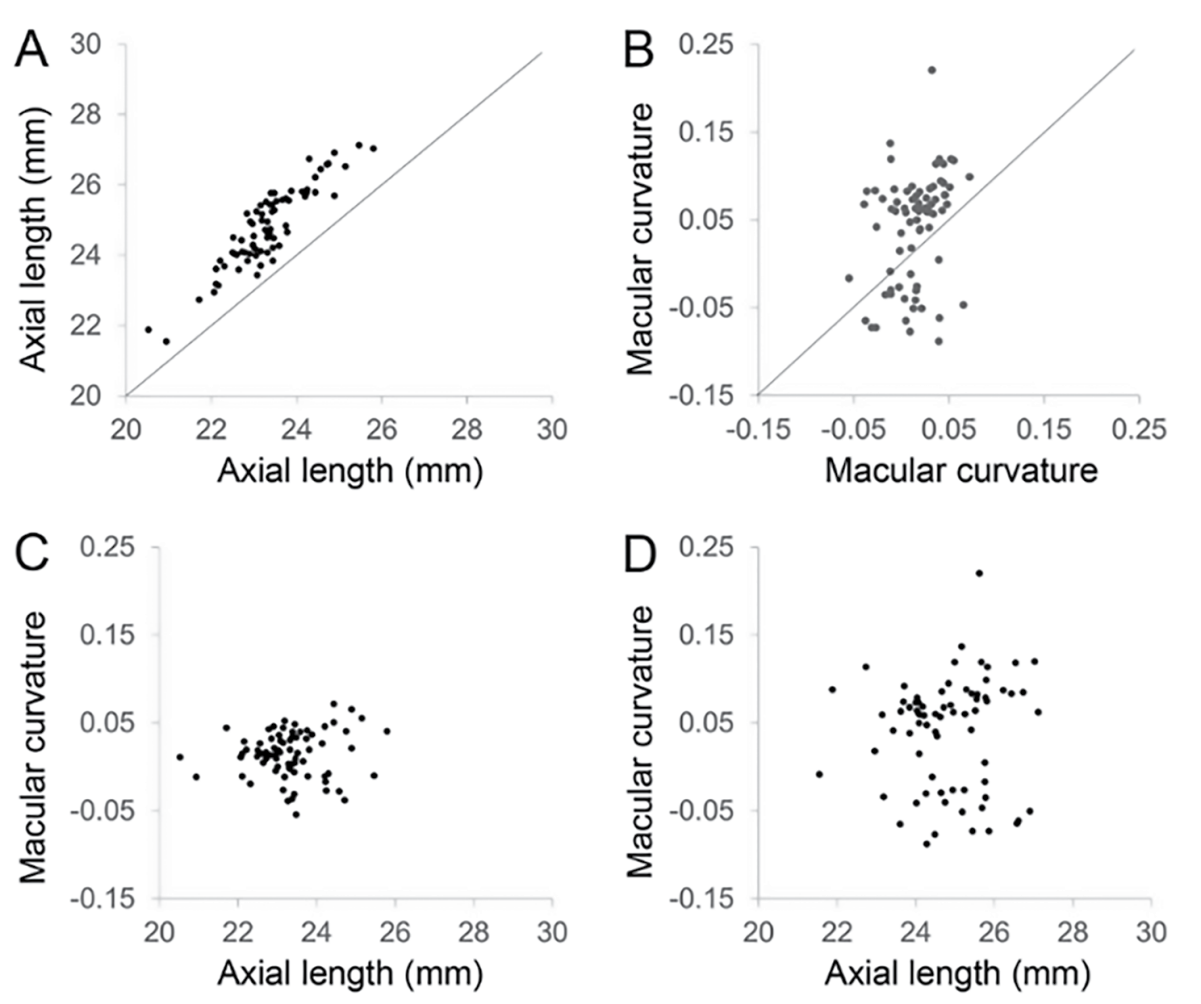
Scatter plots of ocular parameter changes over 6 years in school children. (**A**) Scatter plot of axial length at 14–15 years (vertical axis) versus 8–9 years (horizontal axis), showing a significant increase over time (mean increase: 1.48 ± 0.50 mm; *P* < 0.001). (**B**) Scatter plot of macular curvature at 14–15 years (vertical axis) versus 8–9 years (horizontal axis), indicating a significant increase in steepness over time (mean change: 0.027 ± 0.060; *P* < 0.001). (**C**) Scatter diagram illustrating the relationship between axial length and macular curvature in participants aged 8 to 9 years. (*r* = 0.08, *P* = 0.48). (**D**) Scatter plot showing the correlation between axial length and macular curvature at 14–15 years of age (*r* = 0.13, *P* = 0.28)

A total of 75 children were enrolled in the study, comprising 37 boys and 38 girls. The mean axial length was 23.34 ± 0.92 mm at 8–9 years and 24.82 ± 1.14 mm at 14–15 years. Over the six years, the axial elongation was 1.48 ± 0.50 mm, and the axial length became significantly longer (*P* < 0.001) (Fig. 2A). The mean macular curvature was 0.014 ± 0.026 at 8–9 years and 0.041 ± 0.062 at 14–15 years. Over the six years, the change in macular curvature was 0.027 ± 0.060, and the macular curvature became significantly steeper (*P* < 0.001) (Fig. 2B).

### Association between axial length and macular curvature

**Fig. 3:**
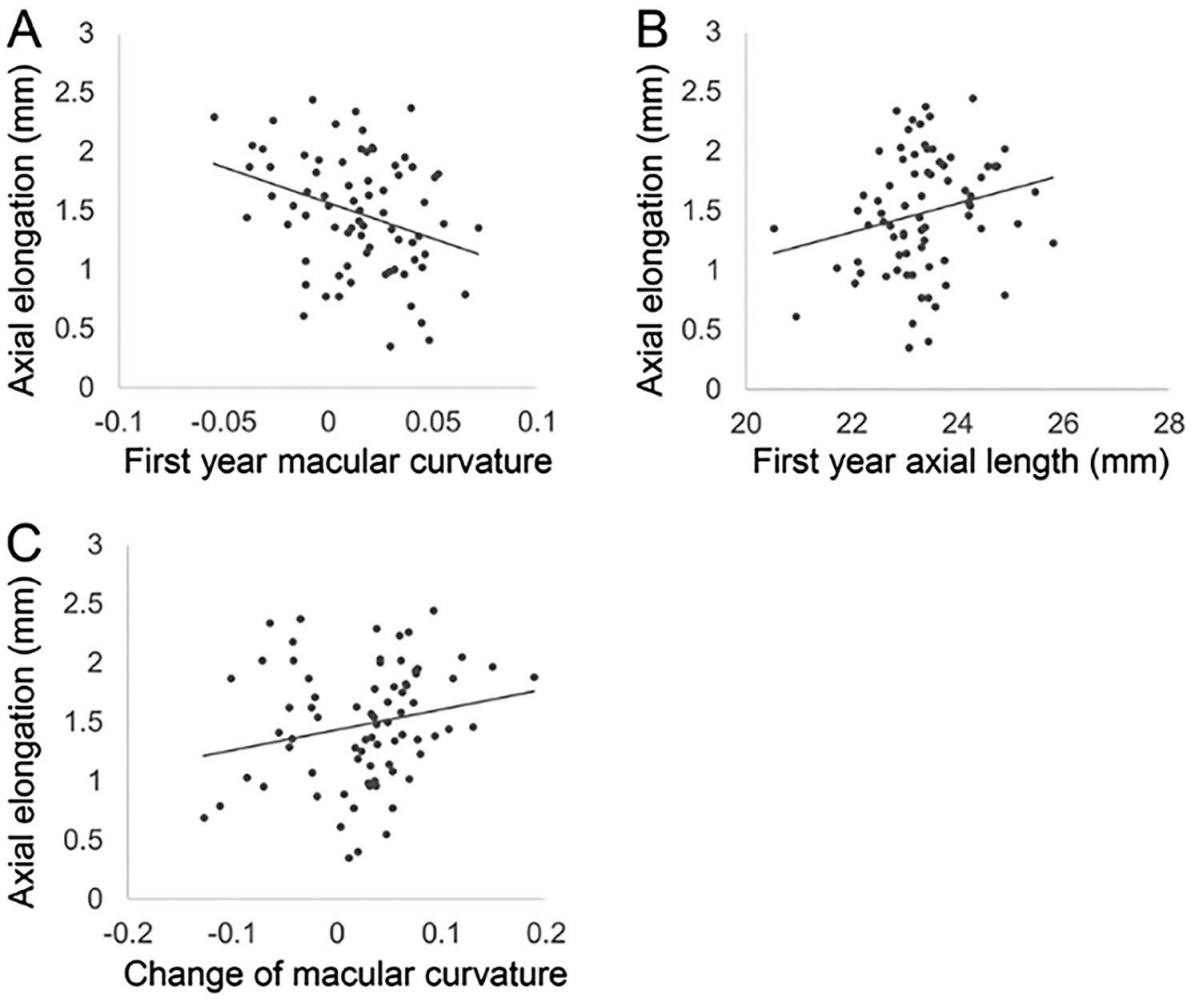
Scatter plots showing the relationship between axial elongation and macular curvature. (**A**) Axial elongation over 6 years was significantly positively correlated with axial length at 8–9 years of age (*r* = 0.23, *P* = 0.046). (**B**) A significant negative correlation was found between macular curvature at 8–9 years and subsequent axial elongation (*r* = − 0.28, *P* = 0.014). Partial correlation analysis after adjusting for baseline axial length also showed a significant association (*r* = − 0.35, *P* = 0.002). (**C**) A significant positive correlation was found between the change in macular curvature and axial elongation over 6 years (*r* = 0.28, *P* = 0.017)

No significant association was observed between macular curvature and axial length at either 8–9 years of age (*r* = 0.08, *P* = 0.48) or 14–15 years of age (*r* = 0.13, *P* = 0.28) (Figs. 2C and D). Axial elongation was significantly positively correlated with axial length in the first year (*r* = 0.23, *P* = 0.046) and macular curvature in the first year (*r* = -0.28, *P* = 0.014) (Figs. 3A, B). Partial correlation analysis after adjusting for first year axial length also revealed a significant correlation between first year macular curvature and axial elongation (*r*= -0.35, *P* = 0.002). Over the six years, the change in macular curvature was significantly positively associated with axial elongation (*r* = 0.28, *P* = 0.017) (Fig. 3C).

**Fig. 4:**
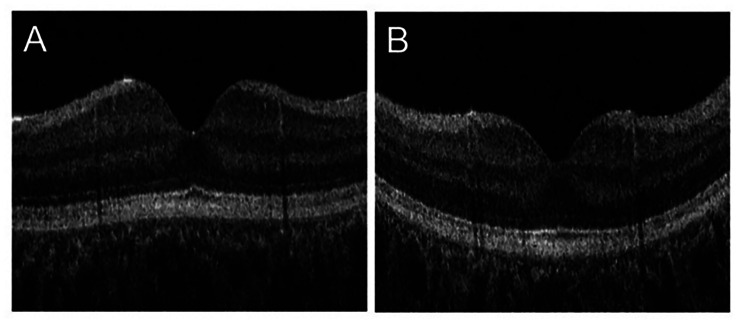
Representative case with increased macular curvature. Macular curvature was steeper at age 8 (**A**) than at age 14 (**B**). The vertical cross-sectional image was vertically quadrupled to make it easier to recognize the shape of the macular area

A representative case of increased macular curvature is shown in Fig. 4. The axial lengths were 22.96 and 24.89 mm, while the macular curvatures were − 0.005 and 0.070 at 8 (Fig. 4A) and 14 (Fig. 4B) years, respectively.

## Discussion

The results showed that the axial length significantly increased between the ages of 8.5 to 14.5 years, and the macular curvature became significantly steeper. Additionally, eyes with steep changes in the macular curvature tended to have greater axial elongation during this period. Steep macular curvature suggests an anteroposterior oval shape of the eye [27, 28]. These results suggest that oval eye enlargement leads to more rapid progression of myopia relative to spherical eye enlargement.

A previous cross-sectional study involving 122 eyes aged 8–9 years and 173 eyes aged 12–13 years reported a significant positive association between macular curvature and axial length in the latter group (*r* = 0.40, *P* < 0.001), whereas no such association was found in the former group (*r* = 0.08, *P* = 0.40) [10]. Eyes with steep changes in the macular curvature tended to have greater axial elongation, but the analysis revealed no meaningful correlation between axial length and macular curvature during both the 8–9 and 14–15 year age periods. The correlation coefficient for axial length and macular curvature was higher at 14–15 than at 8–9 years of age, but the difference was not significant. Therefore, this dissociation in results may be due to the small number of cases in this study.

Both axial length and macular curvature at age 8–9 years were significantly correlated with axial elongation over the following 6 years and are useful as predictors of axial elongation. In addition, there was a significant correlation between first-year macular curvature at age 8–9 years and axial elongation over 6 years adjusted for axial length at age 8–9 years. This suggests that macular curvature at 8–9 years is a predictor of axial elongation independent of axial length at 8–9 years. During this period, eyes with smaller macular curvature at 8–9 years tended to have greater axial elongation. Eyes with smaller macular curvature at age 8–9 years may have more room for convexity and greater axial elongation (Fig. 4).

Fig. 2C, D shows little individual variation in axial length and macular curvature at ages 8–9, but the distribution becomes wider at ages 14–15. Despite the tendency mentioned above, Fig. 2B shows that some eyes have reduced macular curvature during this period, and the change in macular curvature is not uniform.

A previous study showed that eyes with steeper macular curvature were more likely to have longer axial length, lower deep capillary plexus vessel density, and thinner choroidal thickness in children [27]. In adult eyes, high myopia is associated with a lower vessel density [28]. These results suggest that a myopic macula with steeper macular curvature may be under greater mechanical stretching and strain. However, hyperopic children or children with orthokeratology lens wear do not exhibit this phenomenon despite axial length increase [29, 30]. Thus, the mechanisms underlying axial growth in myopia and hyperopia may differ. Two types of eyeball shapes cause the macula to protrude: one is an oval-shaped eyeball from front to back, and the other is macular staphyloma. A slight localized staphyloma will result in a localized tessellated fundus, and focal macular tessellation is not rare in healthy eyes [31]. In this study, macular curvature became significantly steeper with increasing age. Therefore, these irregular ocular enlargements may occur in school-age children. To verify this hypothesis, further studies using OCT, which can capture a wider range of images, are needed.

The primary limitation of this study is its relatively small sample size. Although a minimum of 50 cases is generally considered acceptable for exploratory research [32], a larger sample would likely provide stronger statistical power and reveal more robust correlations between the observed changes. Second, we examined the axial length and not refractive errors. To know the true refractive error in children, it is essential to use cycloplegia with parental consent. However, an agreement was not always obtained; therefore, we focused on the axial length without measuring the refractive error. A third limitation is that we only examined axial length and macular curvature as predictors of axial elongation. Environmental factors and birth factors have been reported as predictors of axial elongation, but these were not taken into consideration in this study. Given the higher prevalence of myopia among Asian populations [30], the generalizability of the present findings to other ethnic groups remains uncertain.

## Conclusion

This cohort study investigated macular curvature changes in healthy children aged from 8 to 9 years to 14–15 years. The axial length significantly increased, and macular curvature became significantly steeper with increasing age. The eyes of children aged 8–9 years with smaller macular curvature tend to have greater axial elongation over 6 years. Macular curvature can be a predictor of axial elongation during this period.

## Data Availability

The datasets used and/or analysed during the current study are available from the corresponding author on reasonable request.

## Abbreviations

OCT: Optical coherence tomography
